# Analysis of the CRISPR-Cas system in bacteriophages active on epidemic strains of *Vibrio cholerae* in Bangladesh

**DOI:** 10.1038/s41598-017-14839-2

**Published:** 2017-11-01

**Authors:** Iftekhar Bin Naser, M. Mozammel Hoque, M. Ausrafuggaman Nahid, Tokee M. Tareq, M. Kamruzzaman Rocky, Shah M. Faruque

**Affiliations:** Laboratory Sciences and Services Division, International Centre for Diarrhoeal Disease Research, Dhaka, 1212 Bangladesh

## Abstract

CRISPR-Cas (clustered regularly interspersed short palindromic repeats-CRISPR-associated proteins) are microbial nuclease systems involved in defense against phages. Bacteria also resist phages by hosting phage-inducible chromosomal islands (PICI) which prevent phage reproduction. *Vibrio cholerae* which causes cholera epidemics, interacts with numerous phages in the environment and in cholera patients. Although CRISPR-Cas systems are usually carried by bacteria and archea, recently *V*. *cholerae* specific ICP1 phages were found to host a CRISPR-Cas system that inactivates PICI-like elements (PLE) in *V*. *cholerae*. We analyzed a collection of phages and *V*. *cholerae* isolated during seasonal cholera epidemics in Bangladesh, to study the distribution, and recent evolution of the phage-encoded CRISPR-Cas system. Five distinct but related phages carrying the CRISPR-Cas system, and possible CRISPR-Cas negative progenitor phages were identified. Furthermore, CRISPR arrays in the phages were found to have evolved by acquisition of new spacers targeting diverse regions of PLEs carried by the *V*. *cholerae* strains, enabling the phages to efficiently grow on PLE positive strains. Our results demonstrate a continuing arms-race involving genetic determinants of phage-resistance in *V*. *cholerae*, and the phage-encoded CRISPR-Cas system in the co-evolution of *V*. *cholerae* and its phages, presumably fostered by their enhanced interactions during seasonal epidemics of cholera.

## Introduction

Bacteriophages contribute to the evolution of their host bacteria in a variety of ways including promotion of genetic diversity by horizontal transfer of genes, and by providing a selective pressure for better fitness of the bacteria^1,2^. *Vibrio cholerae* the causative agent of cholera epidemics interacts with numerous phages in the aquatic ecosystem, and in the intestine of cholera patients. Seasonal epidemics of cholera reportedly collapse due to predation of the pathogen by phages^1,3,4^. Interactions of phage and bacteria may lead to the evolution of numerous phage resistance machineries and mechanisms in bacteria such as blocking of adsorption, abortive infection system, restriction modification system, and the CRISPR-Cas (clustered regularly interspersed short palindromic repeats-CRISPR-associated proteins) system^5–7^. On the other hand, phages also evolve and occasionally effectively bypass these antiviral strategies of bacteria^6,8^.

CRISPR-Cas loci in microbial hosts contain a combination of CRISPR-associated (*cas*) nuclease genes as well as genes for non-coding RNA elements capable of programming the specificity of the CRISPR-mediated nucleic acid cleavage^9,10^. A CRISPR array consists of short direct repeats separated by different short sequences called spacers. To allow nucleic acid cleavage by the associated Cas nuclease, the non-coding CRISPR array is transcribed and cleaved within direct repeats into short crRNAs containing individual spacer sequences. The crRNAs then direct Cas nucleases to the target site (protospacer) which has a sequence complementary to the spacer sequence of the crRNA, and the Cas protein complex degrades the foreign genome in the interference stage^11–13^.

Another way for bacteria to defend against phage infection is by hosting a chromosomal island called phage-inducible chromosomal island (PICI), which being excised from the bacterial chromosome upon phage infection, circularizes, replicates and interferes with phage reproduction^14,15^. The *Staphylococcus aureus* pathogenicity islands (SaPIs), which carry and spread critical virulence genes represent well studied examples of PICIs^14,16^. Upon infection by specific helper phages, SaPIs are induced to excise from the bacterial chromosome, replicate, and package themselves to produce mature phage particles containing SaPI DNA instead of the phage genome, restricting the reproduction of the invading phage^17^. Therefore, following lysis of the infected cell, SaPIs are spread to neighboring cells instead of the helper phage genome. Several strategies used by SaPIs interfere with recognition of the phage genome and its packaging into the phage capsid, and instead promote their own packaging and propagation using the helper phage. These include remodeling the phage capsid proteins to generate small capsids that can accommodate the smaller SaPI DNA and leave out the larger helper phage genome^16^. The SaPIs may encode proteins that interfere with phage packaging by blocking the small subunit of phage terminase, and instead allowing the small subunit of SaPI terminase to bind the phage-encoded large subunit to cleave SaPI DNA for packaging^16^. Another interference mechanism involves interrupting phage late gene activation, which is essential for phage packaging and cell lysis^17^.

*Vibrio cholerae* strains have been found to carry PICI-like elements (PLE) that can resist virulent phages^6,18^. PLE activity was shown to reduce phage genome replication and accelerate cell lysis following infection by ICP1 phages, thus killing infected cells and preventing the production of progeny phage. PLEs were also found to be mobilized by ICP1 infection and spread to neighboring cells^18^.

Strains of the classical biotype of *V*. *cholerae* O1 have been found to carry a CRISPR-Cas system^6,19,20^, which belongs to the previously described type I-E subtype^21^. However, all available genomic sequence data reveal the absence of CRISPR-Cas system in El Tor biotype strains^6^. On the other hand PLEs that respond to infection by ICP1 phages are widespread among *V*. *cholerae*, and consequently PLE mediated inhibition of phage replication is likely to be prominent in *V*. *cholerae* O1 of the El Tor biotype^18^. Although CRISPR-Cas systems are usually carried by bacteria or archea, recently *V*. *cholerae* specific ICP1 phages have been shown to carry a CRISPR-Cas system^6^. Most of the spacer sequences of the CRISPR arrays carried by these phages show 100% sequence identity with different regions of the 18-kb PLE carried by some *V*. *cholerae* O1 El Tor biotype strains^6^. Consequently, the phage encoded CRISPR-Cas system can inactivate the function of PLEs in interfering with phage-reproduction in these strains. The occurrence of a CRISPR-Cas system in cholera phages represents a remarkable event in microbial arms-race and thus warrants further studies to better understand the distribution and evolution of the phage encoded CRISPR-Cas system, and co-evolution of *V*. *cholerae*. Besides, this knowledge would have significance in developing potential phage mediated interventions to control cholera or other bacterial infections.

## Results

### Distribution of CRISPR-Cas system in the phages

**Figure 1:**
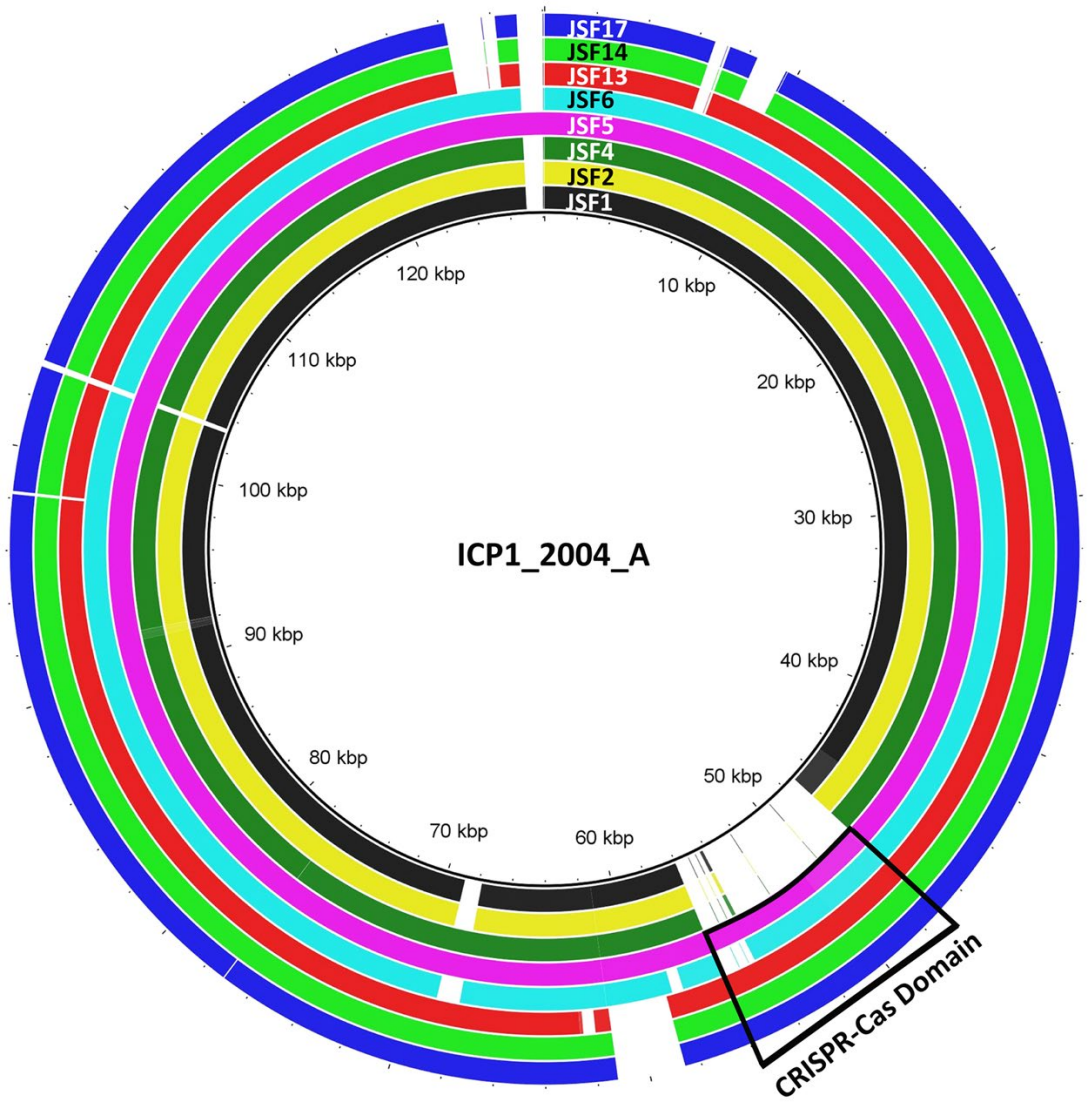
Alignment of the genomic sequences of representative CRISPR-Cas positive and CRISPR-Cas negative phages using a multiple genome alignment software, BRIG^37^. Inside each genomic sequence (shown with colored circles), regions that are absent are shown in white. CRISPR-Cas systems are indicated with a black rectangle. Phages JSF1, JSF2, and JSF4 are CRISPR-Cas negative, but have closely similar genomic sequence to the CRISPR-Cas positive phages (JSF5, JSF6, JSF13, JSF14, JSF17).

**Figure 2:**
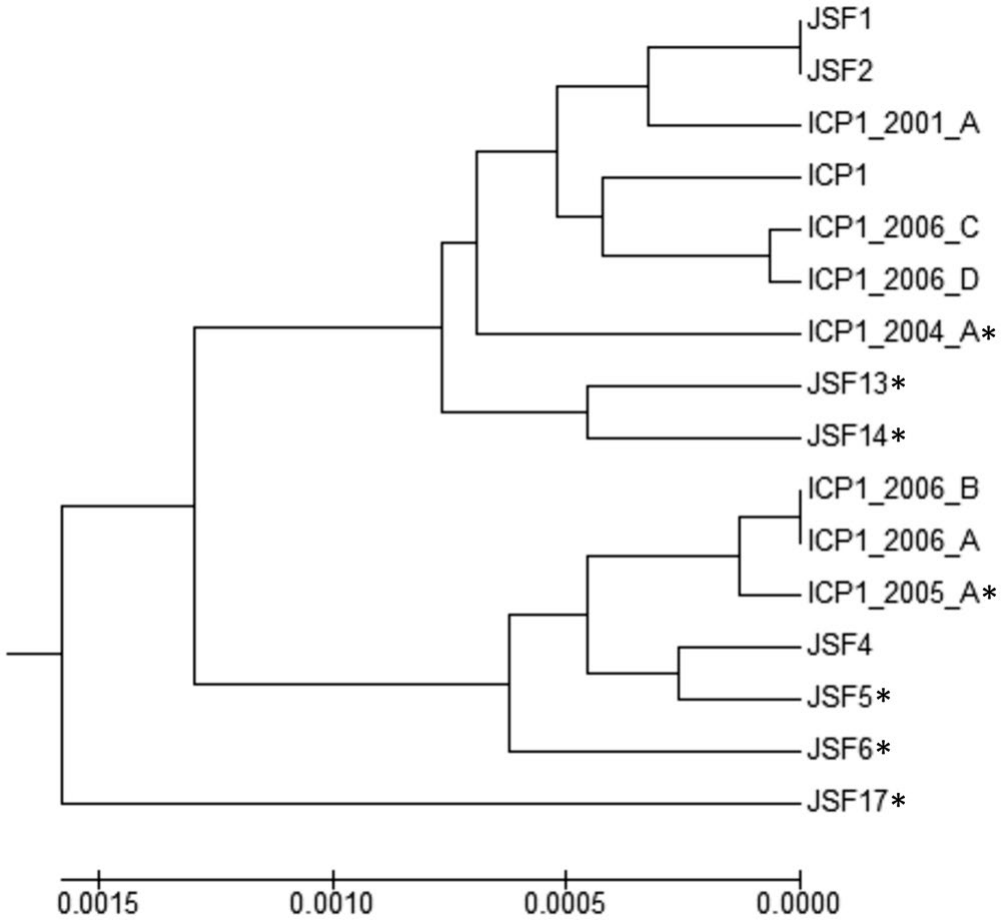
Dendrogram representing phylogenomic relatedness based on nucleotide similarity of whole genomes of 7 CRISPR-Cas positive phages (JSF5, JSF6, JSF13, JSF14, JSF17, ICP1 2004_A and ICP1 2005_A) denoted with an asterisk, and 9 CRISPR-Cas negative phages (JSF1, JSF2, JSF4, ICP1, ICP1 2001_A, ICP1 2006_A, ICP1 2006_B, ICP1 2006_C, and ICP1 2006_D).

*V*. *cholerae* specific phages isolated from environmental waters or stools of cholera patients in Dhaka Bangladesh were initially differentiated based on their host range, and RFLP patterns of their DNA^1,3^. Twenty nine representative phages which were isolated during January 2001 to November 2015, were subjected to whole genome sequencing. Analysis of the genomic sequences for the occurrence and organization of the CRISPR-Cas loci revealed the presence of CRISPR-Cas related sequences in 5 of the 29 phages (17.2%). These 5 phages were related but also had notable differences in their genomic sequence (Fig. 1). We also identified CRISPR-Cas negative phages with otherwise similar genomic sequence to that of the CRISPR-Cas positive phages except for the presence of the CRISPR-Cas loci (Fig. 1), suggesting that the CRISPR-Cas negative phages could possibly be progenitors of the CRISPR-Cas positive phages. On the other hand, since phages also evolve by mosaicism, there may not be any direct progenitor. The genomic sequence of the CRISPR-Cas positive phages excluding the CRISPR-Cas region was between 84% and 99% identical to the whole genome sequences of 9 CRISPR-Cas negative phages (see Supplementary Table S1). The phylogenetic relatedness based on the sequence of the 7 CRISPR-Cas positive phages and 9 CRISPR-Cas negative phages are presented in Fig. 2.

### Diversity of spacers in the CRISPR arrays carried by the phages

**Figure 3:**
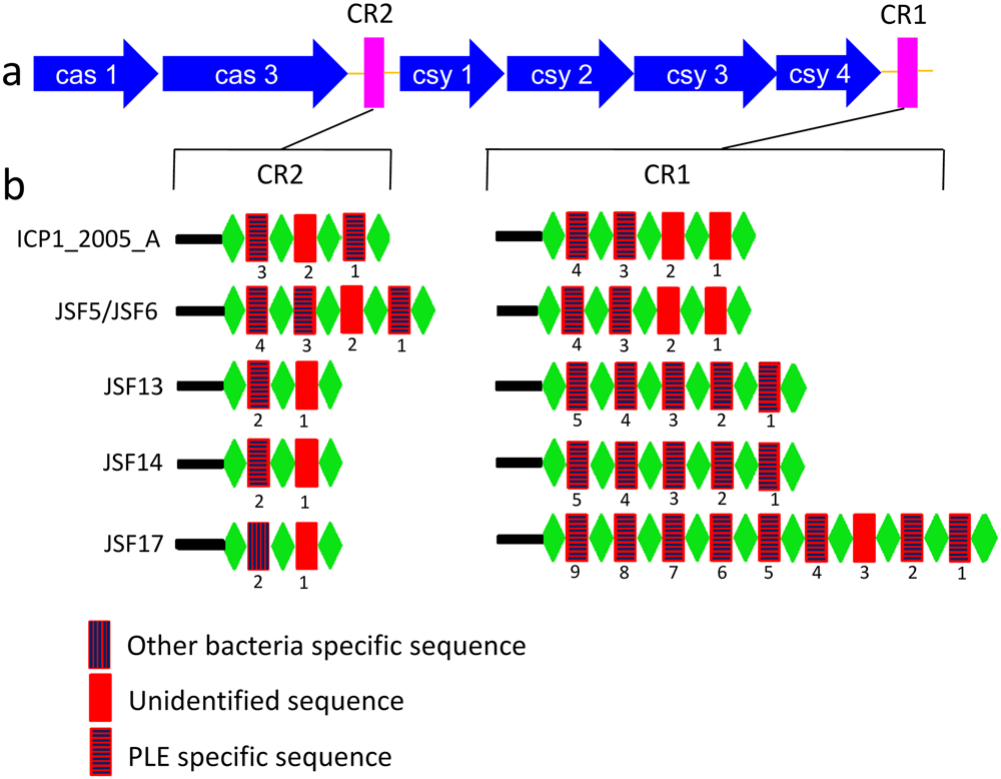
Structure of CRISPR-Cas system carried by phages JSF5, JSF6, JSF13, JSF14, JSF17, and ICP1_2005_A showing the diversity and arrangement of the spacers. The repeat (28 bp) and spacer (32 bp) are shown as green diamonds, and red rectangles respectively. An AT-rich leader sequence precedes each CRISPR locus (black rectangle). Spacers are marked with vertical, horizontal or no lines to show their 100% identity.

**Figure 4:**
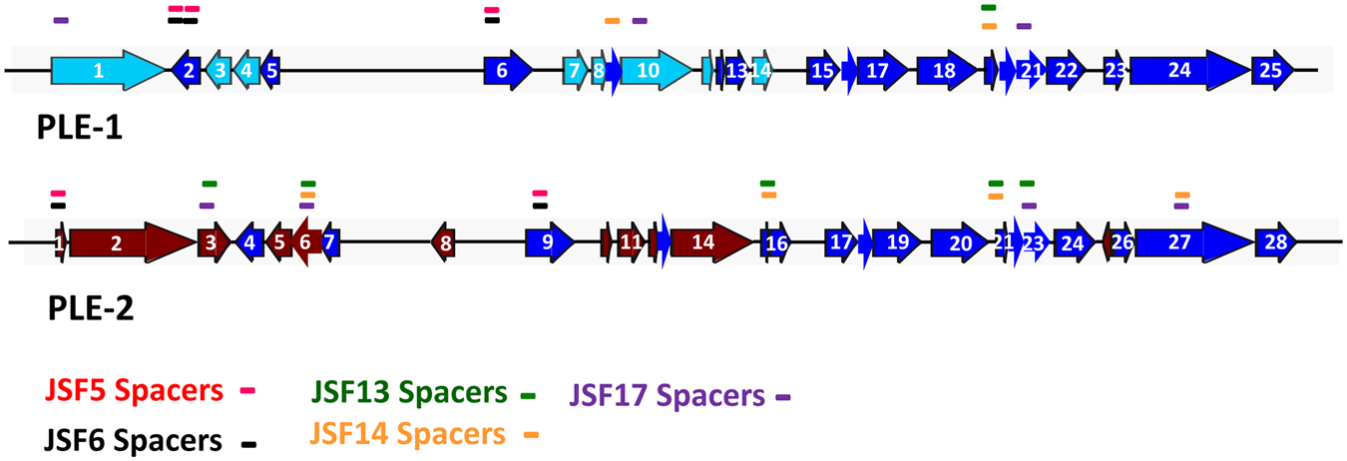
Genomic organization of PLE and protospacer targets of CRISPR-Cas carried by various JSF phages. PLE 1 and PLE 2 consists of 25 and 28 ORFs respectively. Unique ORFs of PLE1 and PLE2 are represented in cyan and fire brick colors respectively. ORFs of PLE1 and the corresponding ORFs of PLE2 that share similar sequences are shown in blue color. The genomic regions of PLE corresponding to spacers of different JSF phages are shown using different colors as indicated.

The overall structure of the CRISPR-Cas loci in the five representative CRISPR-Cas positive phages resembled that of previously reported ICP1 phages^6^, with two CRISPR loci (CR1 and CR2) and 6 *cas* genes (Fig. 3). However, there were differences in the sequences, as well as in number of spacers in the CRISPR array carried by various phages analyzed in the present study. Each of the phages designated JSF5 and JSF6 carried a total of 8 spacers spanning the two CRISPR loci, of which 7 spacers were reported previously in CRISPR carried by ICP1 phages^6^. Although the CRISPR arrays in JSF5 and JSF6 phages were identical, their entire CRISPR-Cas regions were not identical (Fig. 1). Each of JSF13 and JSF14 carried a total of 7 spacers (Fig. 3), but the CRISPR array of JSF13 and JSF14 differed in the sequence of one of the spacers. Three of the spacers of JSF13, and 2 spacers of JSF14 were identical to spacers of ICP1 phages^6^. JSF17 was found to carry a total of 11 spacers and 4 of these were identical to spacers of ICP1 phages. Therefore, 16 of the 33 different spacers identified in the 5 phages were identical to spacers carried by the CRISPR-Cas positive ICP1 phages, whereas the remaining 17 spacers were new. Sequences of most of the spacers in the CRISPR arrays carried by the phages were identical to diverse regions (protospacers) of the two PLEs carried by *V*. *cholerae* strains (Figs 3 and 4) analyzed in our study. We also identified 3 spacers with the corresponding protospacers located in PLE3 reported recently^18^. Notably, all identified protospacers in the PLEs were found to be located within ORFs, and not in intergenic regions. The putative proteins encoded by some of these ORFs found by BLAST or domain analysis are presented in Table S2 (see Supplementary Table S2). Protospacer-adjacent motifs (PAMs) which are short conserved motifs that are present in immediate vicinity of the protospacers were identical to that of previously reported ICP1 phages^6^. Instead of GG PAM present in type I-F CRISPR/Cas system in bacteria^13^ the PAM sequence motifs of the JSF phages analyzed in our study were GA. Sequences of the spacers in the CRISPR arrays and their identity with other DNA are presented in Table 1.

**Table 1:** Sequence of spacers in the CRISPR array carried by different phages.

**Phage**	**CRISPR**	**Spacer**	**Spacer sequence**	Match (100%)*	**Reference Accession No**
JSF 5/6	CR 1	1	TGTGTCTATACTCAACCAATTTAAGCGCCGCA	ICP1_2005_A Spacer^**#**^	HQ641352.1
		2	CTACTCTCCCCAATATTAGCCATTCCTAATTCA	ICP1_2005_A Spacer^**#**^	HQ641352.1
		3	GTCACCTTACCGTAAGACAGGCAGTAAAATTA	PLE 2	KC152961.1
		4	AAACTAGTGGACGTAATGCAGTATTCACGGTT	PLE 1	KC152960.1
	CR 2	1	ATCCACACTACAAATAGAACACTCAACCGTGA	PLE 1	KC152960.1
		2	TATTGATTGGTCTCTAACCTTGGGATGATTAA	ICP1_2005_A Spacer^**#**^	HQ641352.1
		3	AGCGTGTGGGCTTTCATTTTTAAGCCAGTAAA	PLE5	CP001236.1
		4	TTCACGGGTAGCAACAGGGTAATAAACCAATA	PLE 1&2	KC152960.1 KC152961.1
JSF 13	CR 1	1	ATTGCAACTATGCAAAATGATGAAGCTAAAA	PLE3, PLE4	MF176135.1 CP001485.1
		2	GTTAGAGTCGGTAGTATCTGGATGATCGATA	PLE 2, PLE3	KC152961.1 MF176135.1
		3	CACAATCAGCTATAAGCCCTGCATTTTCAAT	PLE 2	KC152961.1
		4	TGTAGTGATGACATAATCTCGTCTCGACTCA	PLE 2	KC152961.1
		5	AGCAGAACTCACCGCCGAAGTGGAACAGCGT	PLE 1&2	KC152960.1 KC152961.1
	CR 2	1	GTGTATTGCTTGCAGTGGGTTACACACAAGA	ICP1_2004_A Spacer^**#**^	HQ641354.1
		2	AAGACGTGACAGCAGTGATCGACTTTATAAC	PLE 2	KC152961.1
JSF 14	CR 1	1	ATTGCAACTATGCAAAATGATGAAGCTAAAA	PLE3, PLE4	MF176135.1 CP001485.1
		2	TCACAATCAGCTATAAGCCCTGCATTTTCAAT	PLE 2	KC152961.1
		3	TTGTAGTGATGACATAATCTCGTCTCGACTCA	PLE 2	KC152961.1
		4	AAGCAGAACTCACCGCCGAAGTGGAACAGCGT	PLE 1 & 2	KC152960.1 KC152961.1
		5	TTGCATCAGTTGGATAGTTAATTGAGTGGGGC	PLE 1	KC152960.1
	CR 2	1	GTGTATTGCTTGCAGTGGGTTACACACAAGA	ICP1_2004_A Spacer^**#**^	HQ641354.1
		2	AAGACGTGACAGCAGTGATCGACTTTATAAC	PLE 2	KC152961.1
JSF 17	CR 1	1	CATTGCAACTATGCAAAATGATGAAGCTAAAA	PLE3, PLE4	MF176135.1 CP001485.1
		2	TGTTAGAGTCGGTAGTATCTGGATGATCGATA	PLE 2, PLE3	KC152961.1 MF176135.1
		3	TTTTGAAACTATTGACAGAAGGTTGGGAACCT	ICP1_2004_A spacer^**#**^	HQ641354.1
		4	TTCAAAATCTTCCGATACATAACTAGCAAGTT	PLE3	MF176135.1
		5	TCACAATCAGCTATAAGCCCTGCATTTTCAAT	PLE 2	KC152961.1
		6	TTGTAGTGATGACATAATCTCGTCTCGACTCA	PLE 2	KC152961.1
		7	AATTGTCGAAGATGGTGAGGCACTAGCTACAC	PLE 1&2	KC152960.1 KC152961.1
		8	TGCGCAGCCACATCACAACACACTGTAAAAAT	PLE 1	KC152960.1
		9	ACAAAACCTTAATAGGGACAAAAGTTATTAAA	PLE 1	KC152960.1
	CR 2	1	GTGTATTGCTTGCAGTGGGTTACACACAAGAA	ICP1_2004_A Spacer^**#**^	HQ641354.1
		2	TTTTACGCAAAGTAGGATCGAGTGTTGCGAAC	*Vibrio cholerae* genomic DNA	CP001485.1

*100% identity was used to designate a spacer as identical to sequence in a PLE or other source.

^#^Denotes the spacer sequences that were found in different phage genomes but their origin are yet to be confirmed.

### Distribution of PLE in *V*. *cholerae* O1 strains

A total of 163 *V*. *cholerae* O1 El Tor biotype strains isolated during 2001 to 2015 were initially screened for the presence of PLE 1 and PLE2 related sequences using PCR assays. All *V*. *cholerae* isolated during 2001 to 2004 were found to be negative for these PLEs. Chronologically, Presence of PLE was first detected in *V*. *cholerae* O1 El Tor strains isolated in 2005. Thereafter, the proportion of strains carrying PLE steadily increased, and all isolates of El Tor *V*. *cholerae* O1 since 2012 were found to carry PLE. Ten representative PLE positive *V*. *cholerae* O1 isolates were subjected to whole genome sequencing, and the sequence of their PLE were compared. Each PLE positive strain carried either one or the other of the two PLE types (see Supplementary Table S3). The sequences of PLE1 and PLE2 were found to be identical to previously published sequences for these two PLEs respectively^18^. Accordingly, PLE1 and PLE2 contained respectively 25 and 28 ORFs, and 17 of these were shared by both PLE1 and PLE2 (Fig. 4). A putative integrase homologue^6^ was encoded by ORF1 in PLE1 and by ORF2 in PLE2. The GC content of PLE1 and PLE2 were 38.3 and 38.6 respectively, which were lower than that of the host *V*. *cholerae* (47.5%).

### Temporal changes in phage-susceptibility of *V*. *cholerae* O1 El Tor strains

**Figure 5:**
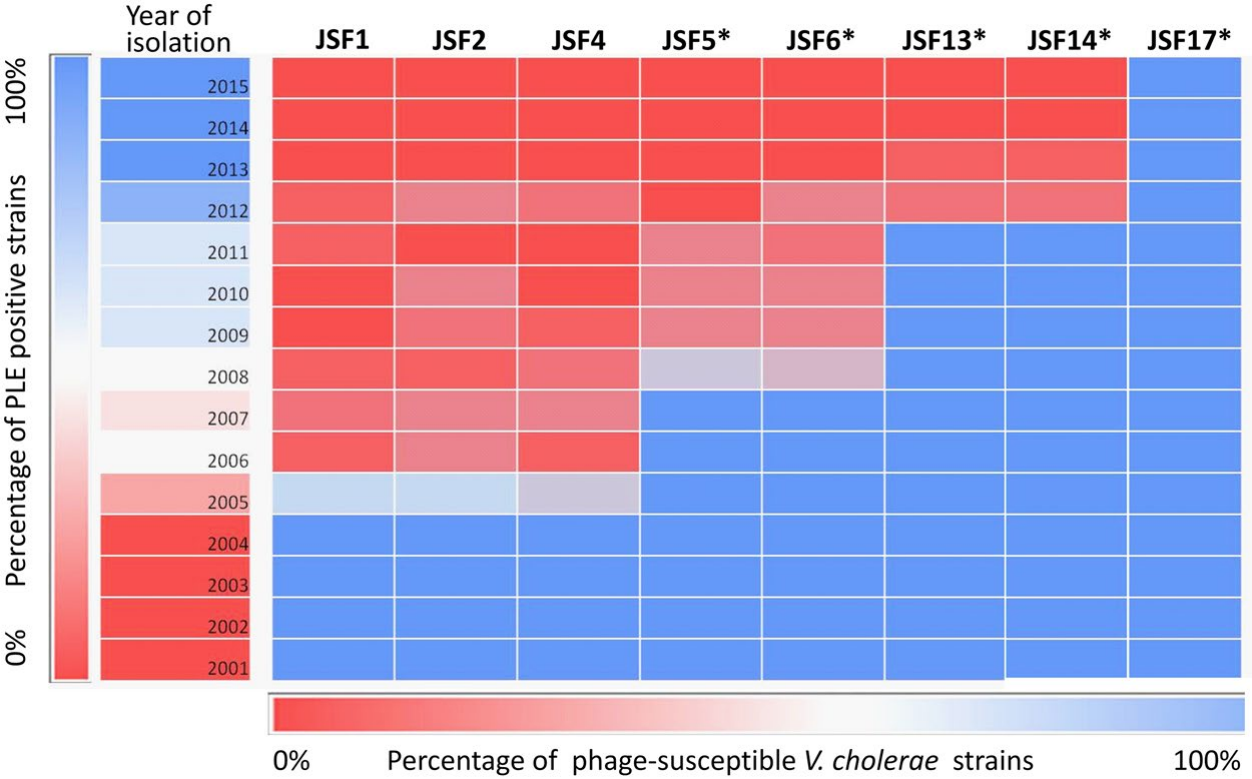
Co-evolution of *V*. *cholerae* O1 and their phages in the PLE versus CRISPR-Cas mediated arms’ race. Heat chart showing the susceptibility of a chronological collection of *V*. *cholerae* O1 isolated during seasonal epidemics of cholera during 2001 through 2015 in Bangladesh to 7 different phages. Phages shown with asterisk are positive for the CRISPR-Cas system.

We analyzed the susceptibility of a chronological collection of *V*. *cholerae* O1 El Tor biotype strains isolated during different cholera epidemics in Bangladesh from 2001 through 2015 to 8 different phages. Five of these phages carried the CRISPR-Cas system whereas 3 were CRISPR-Cas negative, but otherwise similar in genomic sequence to the CRISPR-Cas positive phages (Fig. 1). Further details on these and other phages are available in Table S4 (see Supplementary Table S4). This analysis was done to understand possible effect of the emerging diversity in phage-encoded CRISPR-Cas structures on the susceptibility of *V*. *cholerae* strains with or without harboring the PLE. All *V*. *cholerae* strains isolated during 2001 to 2004 were negative for PLE, and were susceptible to all 8 phages (Fig. 5). Thus the CRISPR-Cas negative phages i.e., JSF1, JSF2, and JSF4 formed clear plaques at high efficiency on the PLE negative *V*. *cholerae* strains isolated during 2001 to 2004. However, with the emergence of PLE2 positive *V*. *cholerae* isolates, the susceptibility of *V*. *cholerae* to the CRISPR-Cas negative phages diminished (see Supplementary Table S3). On the other hand, as expected CRISPR-Cas positive phages represented by JSF5 and JSF6 were able to infect and form plaques on all *V*. *cholerae* isolates from 2001 through 2007 including those that carried PLE2 and were resistant to the CRISPR-Cas negative phages. Similarly JSF13 and JSF14 with more and diverse spacers in the CRISPR array could plaque on most *V*. *cholerae* isolated during 2001 to 2011 including the post-2008 isolates which were positive for PLE1 and were resistant to JSF5 and JSF6 phages. With highest number of spacers in the CRISPR array, JSF17 phage infected and formed plaques on all *V*. *cholerae* O1 El Tor isolates of 2001 through 2015 included in the study (Fig. 5; see Supplementary Table S3). Notably, JSF17 continued to be one of the most prevalent phages from 2013 through 2015.

### Phage susceptibility of *V*. *cholerae* and CRISPR-Cas target sites in the PLEs

We compared the sequence of PLEs carried by various *V*. *cholerae* strains and identified sequences which matched the spacer sequences in the CRISPR array carried by different phages, in an attempt to explain the observed phage-susceptibility patterns of the *V*. *cholerae* strains. In this analysis, we identified multiple regions in both PLE1 and PLE2 that would be potentially targeted by the CRISPR-Cas system of different phages based on the sequence of the spacers (Fig. 4). In all of these instances the PLEs were targeted within one or more ORFs and not in intergenic regions. Phages JSF5 and JSF6 carried spacers which matched with 3 different loci within two ORFs in PLE1; 2 of these 3 loci were also shared by PLE2. Spacer sequences of JSF13 phage correspond to sequences within 5 different ORFs of PLE2 and one ORF of PLE1. Phage JSF14 carried spacers corresponding to sequences located within 4 different ORFs of PLE2 and 2 ORFs of PLE1, whereas JSF17 phage carried spacers matching sequences within 4 different ORFs of PLE2 and 3 ORFs of PLE1. Notably, there have been a temporal increase in number of ORFs of the two PLEs that were targeted by the CRISPR-Cas system carried by different phages. Apparently, this also increased susceptibility of the PLE positive *V*. *cholerae* strains to phages carrying increasing number of spacers targeting the PLEs. Presumably, targeting multiple protospacers in PLEs might have increased the chance of cleaving the PLE DNA and thus more efficiently diminish the function of PLEs. A semi- quantitative estimate of phage susceptibility of various *V*. *cholerae* strains based on plaques formed by different phages are presented in Table S3 (see Supplementary Table S3).

### Presence of CRISPR-Cas system in *V*. *cholerae* non-O1 non-O139 strains

**Figure 6:**
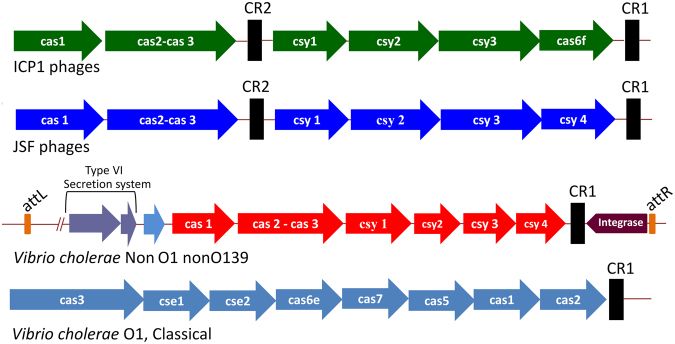
Schematic diagram showing the organization of the CRISPR-Cas loci carried by different cholera phages and *V*. *cholerae* strains. *Cas* genes are shown with colored arrows whereas black rectangles represent the CRISPR locus. The CRISPR-Cas in *V*. *cholerae* non-O1 non-O139 strains are located in a putative transmissible element adjacent to genes for a type VI secretion system. The integrase gene and the attachment sites attL and attR are also shown.

The presence of CRISPR-Cas system has not yet been detected in the El Tor biotype strains of *V*. *cholerae*, although classical strains of *V*. *cholerae* have been reported to carry a CRISPR-Cas system belonging to the I-E subtype^6,19,20^. The CRISPR-Cas found in the classical strains thus belong to a different type than that of the cholera phages, which carry a CRISPR-Cas system belonging to subtype I-F^6,21^. We screened a collection of *V*. *cholerae* non-O1-non-O139 strains for the presence of CRISPR-Cas loci. Of 20 randomly sequenced *V*. *cholerae* non-O1 non-O139 strains, 8 were found to be CRISPR-Cas positive. However, there were wide differences in the nucleotide sequence of different *cas* genes carried by the *V*. *cholerae* non-O1 non-O139 strains and corresponding genes carried by the phages. Primers for *cas1* and *cas3* genes derived from the sequence of the non-O1 non-O139 strains were used in PCR analysis of a further 45 *V*. *cholerae* non-O1 non-O139 isolates, and 23 of these were found to be positive. This data suggested that the occurrence of the CRISPR-Cas system is common among non-O1 non-O139 *V*. *cholerae*. Unlike the phages the CRISPR-Cas locus in *V*. *cholerae* non-O1 non-O139 strains were found to have the features of a genomic island (Fig. 6) as described previously^22,23^.

The CRISPR-Cas region in 7 of 8 CRISPR-Cas positive non-O1 non-O139 strains sequenced in our study were found to be identical with that of strain HC-36A1 described previously^23^. However, comparisons of the entire sequence of the islands in our strains with those of previously described CRISPR-Cas containing genomic islands of strains S12, RC385, RC586 TM 11079-80, and HC-36A1^22,23^, showed that the islands found in 3 of our strains were > 95% identical to that found in strain HC-36A1^23^, whereas the islands in 4 of the strains were 70–74% identical to that in strain HC-36A1. The *cas* genes in one of our strains 173V1015 were found to share no sequence homology with that of the other islands analyzed, although the predicted protein sequence show more than 82% identity with Cas proteins of *Salinivibrio costicola* (Accession no. OOF32727.1, WP_077670175.1) and *Vibrio cholerae* (Accession no. WP_088136878.1, WP_088136879.1, WP_088136862.1), with quarry coverage ranging from 74% to 100%. In all the islands analyzed, Integrase, CRISPR-array and Type VI secretion system genes were found to be conserved with > 95% identity.

## Discussions

In the present study we conducted genomic analysis of representative phages and *V*. *cholerae* strains collected during cholera epidemics in Bangladesh to monitor the distribution and emerging diversity of the CRISPR-Cas system carried by the phages, in view of recurring and extensive phage-bacterial interactions during seasonal epidemics of cholera^1,3^. The unique ecology and socio-economic setting in Bangladesh fostering seasonal cholera outbreaks also provide an opportunity to test predictions into the emergence, co-evolution and diversity of genetic elements involved in the “arms-race” among *V*. *cholerae* and their phages under natural conditions. The CRISPR-Cas system has been described as a microbial adaptive immune system in that the system extends its range of targets by continually acquiring new spacers matching protospacer regions in the invading nucleic acids. The remarkable evolutionary success of the CRISPR-Cas positive phages in countering the PLE mediated defense of *V*. *cholerae* is thus expected to be sustained by further diversification of the CRISPR arrays in terms of number and variety of their spacers.

The nucleotide sequences of the spacers in a CRISPR array carried by the phages should be identical to a region (protospacer) in the target PLE responsible for interfering with reproduction of the invading phage, in order to recognize, and subsequently inactivate the function of the anti-phage system in the bacteria. The genomes of these phages carry a cluster of six *cas* genes and two CRISPR loci, identified as a CRISPR-Cas subtype I-F system^21^. A number of spacers in these CRISPR arrays are identical to sequences within the PLE resident in the *V*. *cholerae* host genome (Fig. 4, and Table 1). Our results further showed that the number of spacers matching the PLEs has progressively increased in phages isolated in different years. Moreover, phages with increased number of spacers were able to form more plaques on PLE positive *V*. *cholerae* strains (see supplementary Table S3). These findings suggest that having more than one spacer targeting the PLE provides increased fitness to the phage in surviving PLE mediated elimination. Since the phages included in this study were isolated during seasonal epidemics of cholera in Bangladesh, these results provide important evidence in support of the adaptive immunity developed by phages through their resident CRISPR-Cas system under natural conditions of phage bacterial interactions, during seasonal cholera epidemics.

Upon infection by ICP1 related phages, the mechanism used by PLEs to resist predation presumably involve excision from the bacterial chromosome, replication, and packaging of PLE DNA into the phage heads, resembling the mechanisms used by *Staphylococcus aureus* pathogenicity islands (SaPIs)^16–18^. Interestingly, we found that all spacers in the phage-encoded CRISPR arrays target various ORFs of the PLE, and not the intergenic regions. While most of these ORFs encode hypothetical proteins of unknown function, we propose that one or more of these gene products may influence the process that restricts phage replication and involves spread of the PLEs instead of the phage genome through heterologous packaging. However, functional studies involving deletion of these target ORFs of PLE would be required to verify this assumption.

*V*. *cholerae* specific phages encoding their own functional CRISPR-Cas system to neutralize a bacterial defense mechanism against phages has been discovered recently^6^, but the origin of the CRISPR-Cas system carried by the phages remains unknown. Mechanisms such as genome rearrangements, and genomic exchange with other viral or microbial genomes to acquire new traits allow phages to evolve rapidly, facilitated by their genomic plasticity and fast multiplication rates. However, the CRISPR-Cas systems carried by *V*. *cholerae* O1 classical biotype strains or *V*. *cholerae* non-O1 non-O139 strains, were found to differ widely from that carried by the phages (Fig. 6). Moreover, the CRISPR-Cas carried by the *V*. *cholerae* non-O1 non-O139 strains were found to be located in a chromosomal island which also carry genes for the Type VI secretion system, as described previously^22,23^. The marked difference at the nucleotide level and absence of features associated with horizontal transfer, suggest that its highly unlikely that the phage encoded CRISPR-Cas was derived from that found in the *V*. *cholerae* non-O1 non-O139 strains. Thus the origin of the CRISPR-Cas locus in cholera phages remains to be identified.

The use of phages as bio-control agents in the environment or in potential phage therapy in patients requires a more clear understanding of the mechanisms causing selection of phage resistant bacteria and the co-evolution of bacteria and phages. The interactions between epidemic strains of *V*. *cholerae* and their lytic phages is known to modulate seasonal epidemics of cholera^1,3,4^. In this process, *V*. *cholerae* undergo genetic modifications to escape phage predation, resulting in a heterogeneous mix of many unique mutants^24^. Thus predatory phages can shape microbial community structure during the natural course of self-limiting epidemics. The acquisition of a CRISPR-Cas system by phages and subsequent evolution of the system to counter bacterial PLE add additional challenges in phage mediated control of cholera. However, monitoring of the CRISPR-Cas arrays in phages, and the bacterial PLE allows to understand the genetic variability and phage-bacterial co-evolution. This knowledge may be useful in designing engineered phages targeting various regions of the bacterial anti-phage genomic determinants, in potential phage therapy or environmental interventions to control cholera.

In summary, we have demonstrated the emerging diversity of the CRISPR-Cas system in cholera phages by acquisition of new spacers to expand their ability to counter PLE-mediated phage defense of diverse *V*. *cholerae* strains. We also showed the presence of a CRISPR-Cas system in a number of *V*. *cholerae* non-O1 non-O139 strains. However, features of the CRISPR-Cas carried by the phages and that of the *V*. *cholerae* non-O1 non-O139 strains differ considerably, and hence do not support a direct relationship in terms of their origin. On the other hand, extensive phage-bacterial interactions during seasonal epidemic cycles of cholera might have contributed to rapid evolution of the CRISPR-Cas system in phages and deviate considerably from the original source. The antagonistic interaction between a genetic determinant of phage resistance in *V*. *cholerae*, and the evolving phage encoded CRISPR that neutralize the bacterial defense, represent a continuing arms’ contest that is occurring between *V*. *cholerae* and its phages. In addition to a better understanding of the evolution of CRISPR-Cas systems in phages, these results may have relevance in developing engineered phages and strategies for phage mediated control of cholera.

## Materials and Methods

### *V*. *cholerae* strains and Phages

*Vibrio cholerae* and phage strains were of environmental or clinical origin and were isolated during different seasonal epidemics of cholera in Bangladesh^1,3,25,26^. These phages were initially selected from numerous isolates based on a preliminary analysis of their DNA restriction patterns and host range^1,25^, and hence represented distinct groups. Phages were isolated from surface water samples in Dhaka city or stools collected anonymously from cholera victims reporting to the hospitals of the International Centre for Diarrhoeal Disease Research, Bangladesh (icddr,b). One or more of the indicator strains including G-3669 (El Tor), P-27457 (El Tor), AI 1852 (O139), and MO1220 (O139) were used as host bacteria for purification and amplification of different phages for the present study. Phages were stored in SM buffer (100 mM NaCl, 8.1 mM MgSO4, 0.05 mM Tris-Cl [pH 7.5], 0.01% gelatin) at 4 °C.

### Plaque assay for detection and quantification of phages

The soft agar plaque assay^27^ was used to detect and estimate phage concentration in samples. Briefly, logarithmic-phase cells (500 µl) of a host bacterial strain in nutrient broth (Difco, Detroit, Mitch.) were mixed with 3.5 ml aliquots of soft agar (nutrient broth containing 0.8% Bactoagar, Difco), and the mixture was overlaid on nutrient agar plates. Samples tested for the presence of phages including aliquots of water, cholera stool supernatants, or bacterial culture supernatants, were pre-filtered through 0.22 μm pore size filters (Millipore Corporation, Bedford, MA) to make them bacteria-free, inoculated on the plates, and incubated for 16 h at 37 °C. A sample was scored positive for phages when a plaque was observed on the bacterial lawn in the plates. Plaques were counted to estimate the concentration of phage particles in the sample.

### Phage production and testing host specificity

A single discrete phage plaque was purified three times by the soft agar (0.7%) overlay method^27^ with a susceptible *V*. *cholerae* strain. For growing the phage in liquid medium, an overnight culture of the host strain was diluted 1:100 in fresh nutrient broth and grown at 37 °C for 4 h. The culture was then inoculated with phages from a single plaque. The bacterium-phage culture was incubated at 37 °C for 16 h, when lysis of most of the bacteria occurred. The culture was centrifuged at 10,000 x *g* for 20 min, and the supernatant was filtered through a 0.22 μm pore size filter (Millipore). The number of phage particles in the filtered supernatant was determined by testing serial dilutions of the supernatant by the soft agar overlay method with the propagating strain. The host range for the phage was tested at a titer of 10^3^ pfu/ml using a variety of bacterial strains. For a semi-quantitative comparison of susceptibility of different bacterial strains to particular phages, susceptibility was scored as −, +, + and +++, based on the number of plaques formed with an equal titer of phage particles used in the soft-agar assay.

### Isolation and analysis of phage nucleic acids

For isolation and analysis of phage nucleic acids, culture supernatants containing phage particles were filtered through 0.22 μm pore-sized filters (Millipore). The filtrates were mixed with one-fourth volume of a solution containing 20% polyethylene glycol (PEG-6000) and 10% NaCl, and centrifuged at 12000 x *g* to precipitate phage particles. The precipitate was dissolved in a solution containing 20 mM Tris-Cl (pH 7.5), 60 mM Kcl, 10 mM MgCl, 10 mM NaCl, and digested with pancreatic DNAseI (100 units/ml) and RNAse A (50 μg/ml) at 37 °C for 2 hours. The solution was extracted with phenol-chloroform, and the total nucleic acids were precipitated with ethanol. Phage nucleic acids were suspended in deionized water and purified using the SV Minipreps DNA purification system (Promega Madison, USA). The phage nucleic acid was digested with restriction endonucleases (Invitrogen Corporation, Carlsbad, CA) and analyzed by agarose gel electrophoresis following standard procedures to initially check for diversity and select different phages for sequencing.

### PCR Assays

Two different PCR assays were used for screening of *V*. *cholerae* strains for the presence of PLE and CRISPR-Cas related sequences. The sequence of primers used for PLE 1 were F-TGCTAGAAGCTGCCAAAGGT, and R-TTGTTGTCCAGCTTCCACTG, and those for PLE2 were F- CAACAGGAATTGCAAGCAGA, and R- CTCCAAACCTGCAAACCATT. Sequence of primers used for *cas1* were F- GCT GGC TCT CAT TCT GGT T, and R- GCT GGC GAA ACT CTT GTT C, and those for *cas3* were F-GCTAAACACCAGCACCACAA, and R- GCGACTTTTCATCCACCAAC. PCR amplification was performed using a Bio-Rad PCR machine, in a 25 ml reaction volume consisting of 12.5 µL Taq master mix, 0.5 µL forward primer, 0.5 µL reverse primer, 10 µL nuclease free water, 0.5 µLDMSO, and 1 µL template DNA. Thermocycle parameters for PLE PCR were 90 seconds at 95 °C for initial denaturation, followed by 35 cycles of 30 sec at 95 °C, 30 sec at 55 °C (primer annealing), and 90 sec at 72 °C; plus a final extension at 72 °C for 5 min. Thermocycle parameters for *cas1 and cas3* PCR were same as above except that the annealing temperature used was 57 °C. Expected sizes of the PCR products were verified by agarose gel electrophoresis with appropriate DNA size markers using standard methods^21^.

### Genome sequencing and analysis

The phage and bacterial genomes were sequenced at the icddr,b genomics centre using Illumina based technology. Genomic fragment libraries for whole-genome sequencing were prepared using Illumina Nextera® XT DNA library Preparation Kit (Cat. no, FC-131-1024) as per manufacturer’s instructions, and sequencing was conducted with Illumina Nextseq. 500 or MiSeq sequencers. FastQC tool was used to check the quality of raw sequence. Sequence reads with average quality less than Q20 were removed using Prinseq-lite v0.20.4^28^. Prinseq was also used to trim end bases from both ends of the reads. *De Novo* assemblies of reads obtained from bacterial and phage genomes were performed using Velvet^29^, or Spade Genome Assembler^30^. Assemblies were further improved by scaffolding with SSPACE v2.0^31^ and gap filling by GapFiller v1.10^32^.

Bacterial *De Novo* assembled sequences were reordered against *V*. *cholerae* N16961 reference genome using progressive algorithm mode of Mauve v2.4.0^33^. Assembled contigs were annotated with the Rapid Annotations using Subsystem Technology (RAST) server^34^ and PROKKA^35^. Comparison and mapping visualization was undertaken using a combination of the software Mauve^33^, Artemis^36^ and BRIG^37^. Bacteriophage *de novo* assembled sequences were searched for homology by BLAST^38^. Multiple bacteriophage sequence alignments were built using MAFFT v.7^39^. From this alignment, UPMGA phylogenetic tree was constructed using MEGA^40^. CRSIPRfinder online tool was used to find out CRISPR sequences in the assembled genomes^41^.

### GenBank accession numbers

The sequences reported in this paper have been deposited in the GenBank database. For a list of accession numbers, see Supplementary Table S4 and Table S5.

### Institutional approvals

All experimental protocols were approved by the Research Review Committee (RRC) and the Ethics Review Committee (ERC) of the icddr,b (Protocol numbers PR-15029 and PR-07018). All methods were conducted in accordance with the guidelines of the RRC and ERC. Informed consent was obtained for using any human sample, as directed in the ERC guidelines.

## Electronic supplementary material

Supplementary information
