# Supplementary material for: Analysis of the CRISPR-Cas system in bacteriophages active on epidemic strains of Vibrio cholerae in Bangladesh
Source: Sci Rep. 2017 Nov 1;7:14880. doi: 10.1038/s41598-017-14839-2 (PMC5665941; doi:10.1038/s41598-017-14839-2)
Supplement: Supplementary file 1 — Supplementary information [file 41598_2017_14839_MOESM1_ESM.pdf]

Supplementary information

Analysis of the CRISPR-Cas system in bacteriophages active on epidemic strains of *Vibrio cholerae* in Bangladesh

Iftekhar Bin Naser, M. Mozammel Hoque, M. Ausrafuggaman Nahid,
Tokee M. Tareq, M. Kamruzzaman Rocky, and Shah M. Faruque*

Table S1-S5

Table S1. Percentage identity of the genomic sequence of CRISPR-Cas positive phages excluding the CRISPR-Cas region with corresponding genomic sequence of three CRISPR-Cas negative phages

CRISPR-Cas positive phage genomes (excluding the CRISPR-Cas regions)	Sequence Identity (%) with different CRISPR-Cas negative phage genomes								
	**ICP1**	**ICP1_2001_A**	**ICP1_2006_A**	**ICP1_2006_B**	**ICP1_2006_C**	**ICP1_2006_D**	**JSF1**	**JSF2**	**JSF4**
ICP1_2004_A	93	94	95	95	93	93	93	93	95
ICP1_2005_A	96	98	99	99	93	93	96	96	98
JSF5	95	97	98	98	95	95	94	94	97
JSF6	98	97	98	98	95	95	98	98	97
JSF13	90	92	93	93	87	87	90	90	91
JSF14	86	88	89	89	84	84	86	86	88
JSF17	89	91	92	92	87	87	89	89	91

Table S2. Results of BLAST and functional domain analysis for information on predicted proteins encoded by various ORFs of PLE1 and PLE2

<table>
<tr><th>PLE</th><th>Annotation Level</th><th>ORF Number</th><th>Database</th><th>Domain_id</th><th>Protein family</th><th>Description</th></tr>
<tr><td rowspan="7">PLE1</td><td rowspan="6">Domain</td><td>ORF-1</td><td>pfam</td><td>PF13408</td><td>Recombinase</td><td>Recombinase zinc beta ribbon domain</td></tr>
<tr><td rowspan="2">ORF-6</td><td>pfam</td><td>PF12802</td><td>MarR_2</td><td>MarR (Multiple antibiotic resistance regulator) Family</td></tr>
<tr><td>pfam</td><td>PF13412</td><td>HTH_24</td><td>Winged helix-turn-helix DNA-binding</td></tr>
<tr><td>ORF-17</td><td>pfam</td><td>PF06391</td><td>MAT1</td><td>CDK (Cyclin dependent kinase)-activating kinase assembly factor MAT1</td></tr>
<tr><td>ORF-20</td><td>pfam</td><td>PF16326</td><td>ABC_tran_CTD</td><td>ABC (ATP-binding cassette) transporter C-terminal domain</td></tr>
<tr><td style="display:none"></td></tr>
<tr><td>Protein</td><td>ORF-2</td><td>Rast</td><td>-</td><td>-</td><td>Phage Protein</td></tr>
<tr><td rowspan="10">PLE2</td><td rowspan="7">Domain</td><td>ORF-6</td><td>pfam</td><td>PF04803</td><td>Cor1</td><td>Cor1/Xlr/Xmr conserved region</td></tr>
<tr><td rowspan="2">ORF-9</td><td>pfam</td><td>PF12802</td><td>MarR_2</td><td>MarR (Multiple antibiotic resistance regulator) Family</td></tr>
<tr><td>pfam</td><td>PF13412</td><td>HTH_24</td><td>Winged helix-turn-helix DNA-binding</td></tr>
<tr><td>ORF-18</td><td>pfam</td><td>PF00196</td><td>GerE</td><td>LuxR-type DNA-binding HTH domain</td></tr>
<tr><td>ORF-19</td><td>pfam</td><td>PF06391</td><td>MAT1</td><td>CDK (Cyclin dependent kinase)-activating kinase assembly factor MAT1</td></tr>
<tr><td>ORF-22</td><td>pfam</td><td>PF16326</td><td>ABC_tran_CTD</td><td>ABC (ATP-binding cassette) transporter C-terminal domain</td></tr>
<tr><td style="display:none"></td></tr>
<tr><td rowspan="3">Protein</td><td>ORF-2</td><td>Rast</td><td>-</td><td>-</td><td>Phage Recombinase</td></tr>
<tr><td>ORF-4</td><td>Rast</td><td>-</td><td>-</td><td>Phage Protein</td></tr>
<tr><td>ORF-8</td><td>Rast</td><td>-</td><td>-</td><td>Mobile element protein</td></tr>
</table>

Table S3. Varying degree of susceptibility of *Vibrio cholerae* O1 strains isolated during 2001-2015 to different phages

Year	Presence and type of PLE		Number of strains with various degree of susceptibility to different phages (indicated by +/- signs)															
			JSF1				JSF2				JSF4				JSF5*			
			+++	++	+	-	+++	++	+	-	+++	++	+	-	+++	++	+	-
2001	PLE negative	10	10	0	0	0	10	0	0	0	10	0	0	0	10	0	0	0
	PLE positive	0																
2002	PLE negative	10	10	0	0	0	10	0	0	0	10	0	0	0	10	0	0	0
	PLE positive	0																
2003	PLE negative	10	10	0	0	0	10	0	0	0	10	0	0	0	10	0	0	0
	PLE positive	0																
2004	PLE negative	10	10	0	0	0	10	0	0	0	10	0	0	0	10	0	0	0
	PLE positive	0																
2005	PLE negative	7	7	0	0	0	7	0	0	0	6	0	0	1	7	0	0	0
	PLE positive	3 (PLE2)	0	0	1	2	0	0	1	2	0	0	1	2	0	3	0	0
2006	PLE negative	4	1	0	0	3	3	0	0	1	1	0	0	3	4	0	0	0
	PLE positive	6 (PLE2)	0	0	0	6	0	0	0	6	0	0	0	6	4	2	0	0
2007	PLE negative	5	2	0	0	3	3	0	0	2	3	0	0	2	5	0	0	0
	PLE positive	5 (PLE2)	0	0	0	5	0	0	0	5	0	0	0	5	2	3	0	0
2008	PLE negative	4	0	0	1	3	1	0	0	3	2	0	0	2	3	0	0	1
	PLE positive	6 (PLE2)	0	0	0	6	0	0	0	6	0	0	0	6	0	0	0	6
2009	PLE negative	3	0	0	0	3	2	0	0	1	1	0	0	2	2	0	1	0
	PLE positive	7 (PLE2)	0	0	0	7	0	0	0	7	0	0	0	7	0	0	0	7
2010	PLE negative	3	0	0	0	3	1	1	1	0	0	0	0	3	2	0	1	0
	PLE positive	7 (PLE2)	0	0	0	7	0	0	0	7	0	0	0	7	0	0	0	7
2011	PLE negative	3	0	0	0	3	0	0	0	3	0	0	0	3	0	0	1	2
	PLE positive	7 (PLE1)	0	1	0	6	0	0	0	7	0	0	0	7	0	0	1	6
2012	PLE negative	1	0	0	1	0	0	0	1	0	0	0	0	1	0	0	0	1
	PLE positive	9 (PLE1)	0	0	0	9	0	0	0	9	0	0	0	9	0	0	0	9
2013	PLE negative	0																
	PLE positive	10 (PLE1)	0	0	0	10	0	0	0	10	0	0	0	10	0	0	0	10
2014	PLE negative	0																
	PLE positive	10 (PLE1)	0	0	0	10	0	0	0	10	0	0	0	10	0	0	0	10
2015	PLE negative	0																
	PLE positive	10 (PLE1)	0	0	0	10	0	0	0	10	0	0	0	10	0	0	0	10

Table S3 continued…

Year	Presence and Type of PLE		JSF6*				JSF13*				JSF14*				JSF17*			
			+++	++	+	-	+++	++	+	-	+++	++	+	-	+++	++	+	-
2001	PLE negative	10	10	0	0	0	10	0	0	0	10	0	0	0	10	0	0	0
	PLE positive	0																
2002	PLE negative	10	10	0	0	0	10	0	0	0	10	0	0	0	10	0	0	0
	PLE positive	0																
2003	PLE negative	10	10	0	0	0	10	0	0	0	10	0	0	0	10	0	0	0
	PLE positive	0																
2004	PLE negative	10	10	0	0	0	10	0	0	0	10	0	0	0	10	0	0	0
	PLE positive	0																
2005	PLE negative	7	7	0	0	0	7	0	0	0	7	0	0	0	7	0	0	0
	PLE positive	3 (PLE2)	2	1	0	0	3	0	0	0	3	0	0	0	3	0	0	0
2006	PLE negative	4	4	0	0	0	4	0	0	0	4	0	0	0	4	0	0	0
	PLE positive	6 (PLE2)	4	2	0	0	6	0	0	0	6	0	0	0	6	0	0	0
2007	PLE negative	5	3	0	1	1	5	0	0	0	5	0	0	0	5	0	0	0
	PLE positive	5 (PLE2)	1	4	0	0	4	1	0	0	4	1	0	0	5	0	0	0
2008	PLE negative	4	4	0	0	0	4	0	0	0	4	0	0	0	4	0	0	0
	PLE positive	6 (PLE2)	0	1	1	4	5	1	0	0	6	0	0	0	6	0	0	0
2009	PLE negative	3	2	0	1	0	3	0	0	0	3	0	0	0	2	0	0	1
	PLE positive	7 (PLE2)	0	0	0	7	5	2	0	0	7	0	0	0	7	0	0	0
2010	PLE negative	3	2	0	1	0	3	0	0	0	3	0	0	0	3	0	0	0
	PLE positive	7 (PLE2)	0	0	0	7	6	1	0	0	7	0	0	0	7	0	0	0
2011	PLE negative	3	0	0	0	3	3	0	0	0	3	0	0	0	3	0	0	0
	PLE positive	7 (PLE1)	0	1	0	6	7	0	0	0	7	0	0	0	7	0	0	0
2012	PLE negative	1	0	1	0	0	0	1	0	0	0	1	0	0	1	0	0	0
	PLE positive	9 (PLE1)	0	0	0	9	0	0	0	9	0	1	0	8	9	0	0	0
2013	PLE negative	0																
	PLE positive	10 (PLE1)	0	0	0	10	0	0	0	10	0	0	0	10	10	0	0	0
2014	PLE negative	0																
	PLE positive	10 (PLE1)	0	0	0	10	0	0	0	10	0	0	0	10	10	0	0	0
2015	PLE negative	0																
	PLE positive	10 (PLE1)	0	0	0	10	0	0	0	10	0	0	0	10	10	0	0	0

All *V. cholerae* El Tor strains analyzed were negative for the CRISPR-Cas system. Phages positive for the CRISPR-Cas system are marked with an asterisk.

Table S4. Details of phages sequenced in this study

Phage	Year of isolation	source	GenBank Accession no
JSF1	2001	Water	KY883636
JSF2	2001	Water	KY883637
JSF3	2002	Water	KY065148
JSF4	2002	Patient	KY065147
JSF5	2002	Water	KY883634
JSF6	2002	Water	KY883635
JSF7	2005	Water	KY065149
JSF9	2005	Water	KY883656
JSF10	2007	Water	KY883654
JSF11	2007	Water	KY883641
JSF12	2009	Water	KY883655
JSF13	2009	Water	KY883638
JSF14	2011	Water	KY883639
JSF15	2011	Water	KY883642
JSF17	2012	Water	KY883640
JSF18	2012	Water	KY883650
JSF20	2012	Water	KY883651
JSF23	2012	Water	KY883657
JSF24	2012	Water	KY883652
JSF25	2013	Water	MF574151
JSF27	2013	Water	KY883658
JSF28	2013	Water	KY883643
JSF30	2013	Water	KY883644
JSF31	2014	Water	KY883645
JSF32	2014	Water	KY883646
JSF33	2014	Water	KY883647
JSF34	2015	Water	KY883653
JSF35	2015	Water	KY883648
JSF36	2015	Water	KY883649

Table S5. GenBank Accession numbers of deposited *Vibrio cholerae* genomic sequences

**Organism**	**Strain ID**	**Accession No**
V. cholerae non-O1 non-O139	MGL845	NOJU00000000
V. cholerae non-O1 non-O139	195V0316	NOJV00000000
V. cholerae non-O1 non-O139	4119	NOJH00000000
V. cholerae non-O1 non-O139	4874	NOJI00000000
V. cholerae non-O1 non-O139	173V1015	NOJJ00000000
V. cholerae non-O1 non-O139	MGL390	NOJK00000000
V. cholerae non-O1 non-O139	MGL788	NOXQ00000000
V. cholerae non-O1 non-O139	MGL2619	NOXR00000000
V. cholerae O1 El Tor	2690410	NOJL00000000
V. cholerae O1 El Tor	2736344	NOKR00000000
V. cholerae O1 El Tor	2779947	NOJM00000000
V. cholerae O1 El Tor	2788878	NOJN00000000
V. cholerae O1 El Tor	2924281	NOJO00000000
V. cholerae O1 El Tor	MGL155	NOJP00000000
V. cholerae O1 El Tor	MGL306	NOJQ00000000
V. cholerae O1 El Tor	MGL35	NOJR00000000
V. cholerae O1 El Tor	456300	NOJS00000000
V. cholerae O1 El Tor	471352	NOJT00000000
